# Posttraumatic stress disorder symptoms and coping with the lockdown among help-seeking veterans before and during the COVID-19 pandemic

**DOI:** 10.3325/cmj.2021.62.241

**Published:** 2021-06

**Authors:** Marina Letica-Crepulja, Aleksandra Stevanović, Jasna Grković, Ika Rončević-Gržeta, Nikolina Jovanović, Tanja Frančišković

**Affiliations:** 1 Department of Psychiatry and Psychological Medicine, Faculty of Medicine, University of Rijeka, Rijeka, Croatia; 2 Unit for Social and Community Psychiatry, Bart’s and London School of Medicine and Dentistry Queen Mary University of London, WHO Collaborating Centre for Mental Health Services Development, London, United Kingdom

## Abstract

**Aims:**

To compare the severity of posttraumatic stress disorder (PTSD) symptoms and of particular PTSD clusters among help-seeking veterans before and during the COVID-19 lockdown. The second aim was to identify the main coping strategies used.

**Methods:**

Male war veterans (N = 176) receiving outpatient treatment at the Referral Center for PTSD were assessed at baseline (12-18 months before the pandemic declaration in March 2020) and during the COVID-19 pandemic lockdown (March-June 2020). The Life Events Checklist for DSM-5, PTSD Checklist for DSM-5, and The Brief COPE were used.

**Results:**

Direct exposure to the virus in our sample was low, and the majority of participants followed the preventive measures. The severity of the overall PTSD symptoms and of clusters of symptoms significantly decreased compared with the first assessment. At the second assessment, all participants still fulfilled the PTSD diagnosis criteria. During the lockdown, the participants used emotion-focused and problem-focused coping rather than dysfunctional coping.

**Conclusion:**

The severity of PTSD symptoms decreased during the lockdown. Further research is needed to study the trajectories of long-term psychopathology.

The COVID-19 pandemic has severely threatened the physical and mental health of individuals around the world. Stressors have included isolation, self-isolation or quarantine, restricted movement and physical contact, infection fears, loss of loved ones, lack of supplies, inadequate information (“infodemic”), financial loss, and social stigma (1-3). During emergencies, mental health requires special consideration due to increased rates of stress-related mental health problems and limited availability of mental health services (2-5).

Depending on the emergency context, particular groups of people are at an increased risk of experiencing social and psychological problems (6), and ex-combatants have been repeatedly shown to be one of them (7).

Exposure to a new traumatic or stressful life event might affect posttraumatic stress disorder (PTSD) symptoms. A growing body of research shows that such exposure is a risk factor for worsening of the condition in various groups of PTSD patients (8,9). This mainly happens if the subsequent event is of the same type as the initial stressors, serving as a reminder and as an additional traumatic factor with a “wear and tear” effect on the exposed person (10-13).

Regarding the coping strategies used among PTSD patients, combat veterans with PTSD report a more ineffective and dysfunctional coping style, with the avoidance coping style as a predictive factor of the overall PTSD symptom severity (14-16). PTSD patients try to avoid confrontation with trauma-reminders, intrusive memories, and trauma-related thoughts and emotions (17,18). Some authors emphasize the importance of differentiating between coping strategies with PTSD symptoms and coping strategies with actual traumatic or stressful events as they depend on various factors related to the specific traumatic exposure (19).

Twenty-five years after the Homeland War in Croatia (1991–1995), veterans still suffer from numerous health problems and have been highly prevalent among the users of the health facilities for PTSD treatment (20-22). The same is true for PTSD patients treated in the Referral Center of the Ministry of Health of the Republic of Croatia (RCPTSD) at the Clinical Hospital Center (CHC) Rijeka. A recent study revealed high rates of overall symptoms and severe posttraumatic symptoms (ie, complex PTSD) in this population years after the war ended (23).

The COVID-19 pandemic lockdown in Croatia started on March 19, 2020. According to Oxford University, Croatia introduced the world's strictest restrictions in relation to the number of the infected (24,25). On March 21, 2020, mental health experts from RCPTSD recommended self-help strategies for staying in good mental health and advice for front-liners on how to deliver psychological first aid (26,27). On the same day, the Croatian Psychiatric Association Expert Group released recommendations for the organization of psychiatric care, psychiatric interventions, and psychopharmacological treatment of mental conditions during the COVID-19 pandemic, and for de-escalation and appropriate communication techniques with aggressive patients (28-30). In RCPTSD, mental health service was restructured to be delivered via the internet or hotlines during the pandemic, with a possibility for urgent outpatient and inpatient treatment. On April 27, 2020, the lifting of restrictions began.

To our knowledge, no study worldwide has examined the psycho-social correlates of the COVID-19 pandemic lockdown in veterans with PTSD. Therefore, we aimed to compare the severity of PTSD symptoms and of particular PTSD symptom clusters before and during the COVID-19 pandemic lockdown. Second, we identified the main coping strategies that veterans used during the lockdown.

## Methods

### Participants and procedures

*The first assessment.* The study design was previously described (31). The original research was conducted from November 2018 to February 2019 and consisted of a structured clinical interview and self-report questionnaires. The participants were male Homeland War veterans with PTSD who had experienced at least one war-related traumatic event defined in the Diagnostic and Statistical Manual of Mental Disorders, Fifth Edition (DSM-5) criteria for PTSD (combat or exposure to a war-zone personally experienced) (32). Participants were considered eligible if they met the diagnostic criteria for war-related PTSD as defined in the DSM-5 (32). From the original sample (n = 300), only participants from RCPTSD at CHC Rijeka were included in the follow-up study.

*The second assessment*. Male war veterans with PTSD referred to RCPTSD assessed in the first phase of the study (n = 250) were approached for the purposes of the second assessment. The authors used the psychological assessment administered in the first phase, even though the general design and aims of the initial research differed from those of the current study. In total, 181 male war veterans with PTSD were re-assessed from April 15 to the end of May 2020 (ie, during and in the immediate aftermath of the COVID-19 pandemic lockdown). Due to the ongoing anti-pandemic restrictions and recommendations, the assessments were mainly conducted by telephone or face-to-face during one or two sessions with participants who attended the check-up. The evaluation consisted of a structured clinical interview and self-report questionnaires. The interviews were conducted by three psychiatrists (MLC, JG, IRG) and a psychologist (AS). A structured interview was created for this study and included questions on demographic data, war-deployment duration, history of psychiatric care, COVID-19-related history, and an open-ended question about the most challenging experience related to the lockdown. After the participants were given detailed information about the study, all participants provided written informed consent. The study was approved by the Ethics Committee of the CHC Rijeka.

### Measures

At both study points, we employed The Life Events Checklist for DSM-5 (LEC-5) and PTSD Checklist for DSM-5 (PCL-5). The Brief COPE was applied only at the second study point.

*The Life Events Checklist for DSM-5*. LEC-5 was used to assess possible lifetime traumatic events experienced by participants (33). The self-report measure lists 16 traumatic events and an additional item indicating any other stressful event. For the study, the total score of lifetime trauma, calculated as the sum of traumatic events, ranges from 0 to 17. The checklist was reported to have good psychometric properties (33,34).

*PTSD Checklist for DSM-5 (PCL-5) with Criterion A*. The PCL-5 with Criterion A is a self-report measure revised to match the adapted DSM-5 criteria for PTSD (35). A provisional PTSD diagnosis can be made considering items rated 2 = moderately or higher according to the DSM-5 diagnostic rule (at least one B, one C, two D, and two E symptoms present). Symptom severity is calculated as the sum of all items (0-80) or as the sum within a specific cluster of symptoms. Validation studies showed excellent psychometric properties for evaluating PTSD (35-38). Cronbach alphas in our study ranged from 0.72 to 0.85 for clusters and 0.90 for total PCL-5.

*The Brief COPE*. The Brief COPE is a 28-item multidimensional measure of coping strategies used for regulating cognitions and behaviors in response to stressors (39). Fourteen two-item scales are rated on the four-point rating scales (1 = I have not been doing this at all to 4 = I have been doing this a lot). The score is the sum of two items. Each scale can be viewed independently or as part of emotion-focused, problem-focused, or dysfunctional coping strategies (39,40). An alternative classification differentiates between adaptive and maladaptive coping (40,41). The Brief COPE is reported to have good psychometric properties (39,42). The Cronbach alpha in our study was 0.81.

### Analysis

Descriptive statistics were used to present the frequencies/percentages or means and standard deviations for parametric measures. The Pearson χ^2^ was used to assess the differences between the groups on categorical variables. In the cases with cells with count <5, the Yates correction was applied. Most of the continuous variables did not meet the normality of distribution criteria (as tested with the Kolmogorov-Smirnov and Shapiro Wilk test), implying the use of non-parametric tests. However, as the Mann-Whitney test and the Wilcoxon signed-rank test yielded the same significances between groups and measurements, their parametric equivalents are reported. Hence, to test the between-group differences for continuous variables, we used the *t* test for independent samples. To assess the differences between two time points for continuous measurements, the *t* test for repeated measures was used. Statistical significance level was set at *P* < 0.05. The analysis was performed with Statistica software, version 12 (Dell Inc. Inc., Tulsa, OK, USA).

## RESULTS

### Demographics and war-related characteristics

Out of 250 participants at the first assessment, 176 (70.4%) participated in the second assessment. Sixty-nine participants were missing – five refused to participate and 64 were unreachable. Five participants were excluded from further analysis due to incomplete data. Sociodemographic characteristics from the first assessment are presented in Table 1. Missing participants and the participants who participated in the second assessment did not differ significantly in any of the sociodemographic characteristics or war-related measures. Therefore, functional equivalent and representativeness allowed further analysis.

**Table 1:** Sociodemographic characteristics of study participants at the first assessment. Data are presented as count (percentage) unless otherwise indicated

	The first-assessment participants, N = 250		Statistics	
	assessed at the second assessment, N = 176	missing at the second assessment, N = 74		
Age*	52.75 (6.04)	51.45 (5.42)	*t* = 1.589	*P* = 0.113
Educational level				
elementary school	22 (12.2)	7 (10.6)	χ^2^ = 5.49	*P* = 0.064
high school	139 (76.8)	57 (86.4)		
higher education	19 (10.6)	1 (1.5)		
Work status				
employed	48 (26.5)	24 (36.4)	χ^2^ = 2.69	*P* = 0.261
unemployed	28 (15.5)	10 (15.2)		
retired	105 (58.4)	31 (47)		
Marital status				
married/cohabitating	129 (71.3)	45 (66.2)	χ^2^ = 3.51	*P* = 0.321
single	26 (14.4)	7 (10.3)		
divorced	18 (9.9)	10 (14.7)		
other	8 (4.4)	6 (8.8)		
Economic status (self-reported)				
high	6 (3.4)	0 (0)	χ^2^ = 5.66	*P* = 0.23
medium	107 (60.1)	47 (71.2)		
low	65 (36.5)	19 (28.8)		
Treatment duration (in years)*	16.28 (8.58)	14.03 (8.02)	*t* = 1.759	*P* = 0.080
Deployment duration (in months)*	31.12 (19.99)	28.06 (17.17)	*t* = 1.252	*P* = 0.212
Life events (LEC-5^†^)*	9.89 (4.53)	9.07 (3.19)	*t* = 1.249	*P* = 0.213

*mean ± standard deviation.

†LEC-5 – The Life Events Checklist for DSM-5.

### COVID-19 related characteristics

Out of the total number of participants at the second assessment (N = 176), one participant was diagnosed with COVID-19 with mild symptoms. Close persons of two participants and acquaintances of three participants were infected with SARS-CoV-2. Two veterans were self-isolated. One participant's family member and two participants' acquaintances were self-isolated.

The majority of the participants said that during the lockdown they had entirely followed (65.7%) or mostly followed (24.7%) the government-imposed COVID-19 restrictions. Most participants did not seek psychiatric assistance besides their pre-scheduled regular clinical meetings (82.4%). Out of 31 (17.6%) participants who did seek additional psychiatric help, 30 did so through telephone consultations, and one had an in-person ambulatory appointment. The majority of participants who sought psychiatric consultations had no change in the treatment plan, and two had medicaments correction.

When asked what the most difficult experience during the lockdown was, a large proportion of war veterans (48.3%) could not single out anything specific. Out of 91 (51.7%) participants who did, 46.2% singled out movement constrictions (not being able to go out, to travel, issues with travel permissions, etc), 34.1% singled out social isolation, 13.2% singled out fear for their family members, 12.1% singled out fear of contracting the virus, and 8.8% singled out existential fears. In total, 14.3% of participants reported a combination of the two most stressful experiences regarding COVID-19 and anti-pandemic measures.

### PTSD symptom severity

Compared with the first assessment, the intensity of the overall PTSD symptoms and all four clusters of symptoms were significantly lower at the second assessment (Table 2).

**Table 2:** Overall PTSD symptom and symptom cluster severity before and during the COVID-19 pandemic lockdown*

	The first assessment		The second assessment			
	range	mean (SD)	range	mean (SD)	t	p
Cluster B symptoms	5-20	14.8 (3.17)	2-20	13.19 (4.55)	4.868	<0.001
Cluster C symptoms	1-8	6.11 (1.47)	0-8	5.78 (1.81)	2.026	0.044
Cluster D symptoms	3-32	21.31 (5.93)	1-29	16.62 (6.63)	9.002	<0.001
Cluster E symptoms	5-24	17.36 (4.08)	2-24	13 (5.17)	10.423	<0.001
PCL-5 total score	18-80	56.8 (11.26)	15-76	46.66 (13.60)	9.416	<0.001

*Abbreviations: PTSD – posttraumatic stress disorder; SD – standard deviation; PCL-5 – PTSD Checklist for DSM-5 (PCL-5) with Criterion A.

To control for the effect of the treatment between two assessments on the overall PTSD symptom severity, participants were divided in three groups: those treated as usual (outpatient treatment including regular appointments and continuous psychopharmacological treatment), those who received inpatient treatment, and those taking part in outpatient day-hospital program for PTSD (Table 3).

**Table 3:** Overall posttraumatic stress disorder (PTSD) symptom severity before and during the COVID-19 pandemic lockdown between two assessments in three treatment groups of PTSD patients

	The first assessment (PCL-5 total score)	The second assessment (PCL-5 total score)		
	mean (SD)	mean (SD)	z	p
Inpatient treatment, n = 16	60.94 (10.21)	48.6 (15.74)	-2.644	0.008
Outpatient treatment – day-hospital, n = 12	55.8 (9.33)	51 (10.8)	-1.768	0.077
Outpatient treatment, treated as usual, n = 148	56.4 (11.45)	46.12 (13.53)	9.002	<0.001

*Abbreviations: SD – standard deviation; PCL-5 – PTSD Checklist for DSM-5 (PCL-5) with Criterion A.

For the inpatient and outpatient check-up groups, the severity of overall PTSD symptoms significantly decreased between the two assessments. The level of overall PTSD symptoms in the outpatient day-hospital program for PTSD decreased but not significantly. All participants fulfilled the criteria for the PTSD diagnosis at both assessments. Treatment groups did not differ considerably in age, treatment duration in terms of the time passed since the first referral, or in the number of traumatic events.

### Coping with COVID-19 related issues

**Figure 1:**
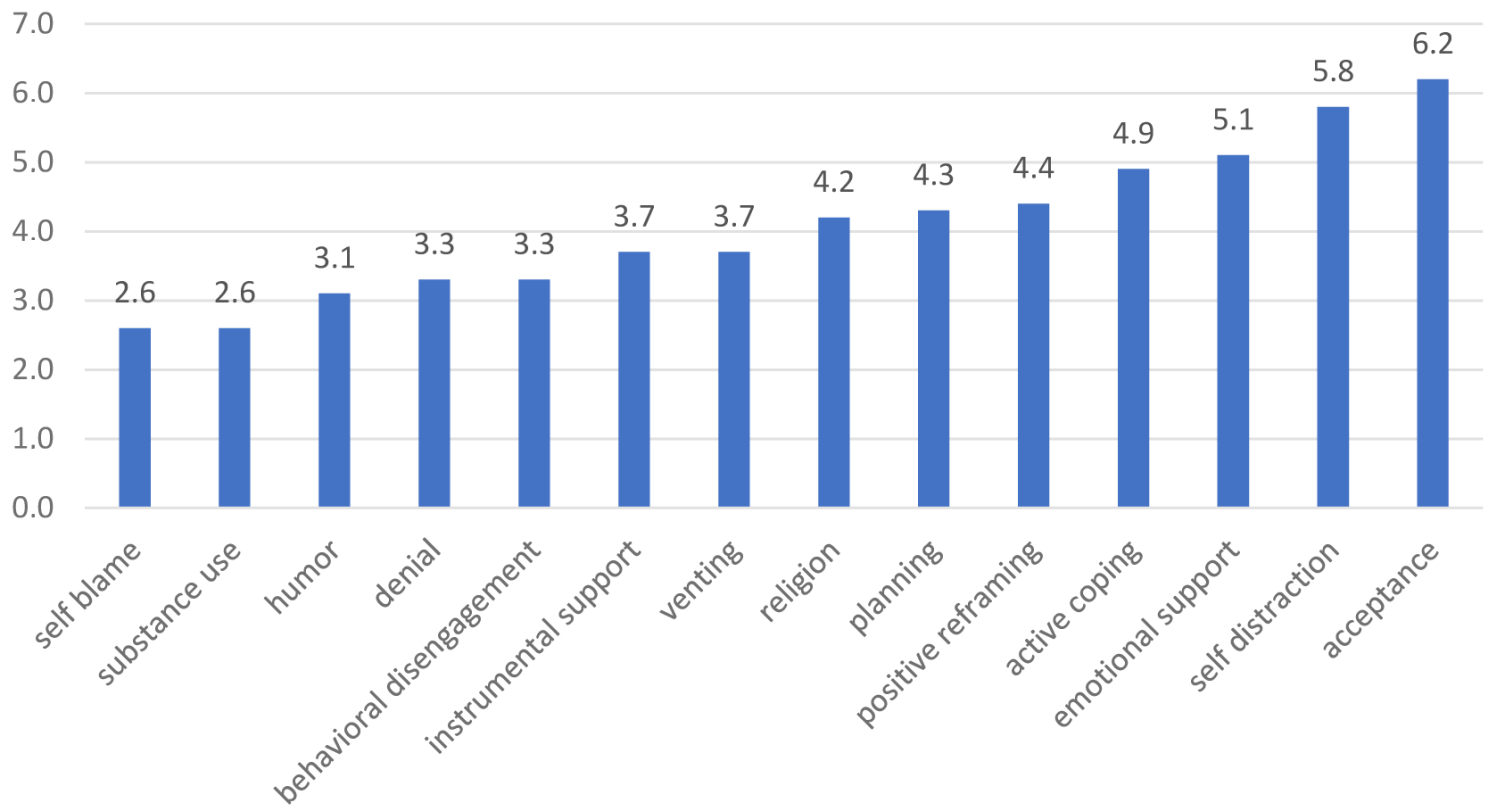
The average score on coping strategies (The Brief COPE).

The coping strategies used during the COVID-19 pandemic that obtained the highest scores were acceptance, self-distraction, and use of emotional support. Substance use and self-blame obtained the lowest scores (Figure 1). In the alternative way of grouping coping strategies, veterans scored higher on emotion-focused coping (X = 4.61, SD = 1.04) and problem-focused coping (X = 4.29, SD = 1.39) than on dysfunctional coping (X = 3.53, SD = 0.83). They scored higher on adaptive coping strategies (X = 4.49, SD = 1.04) compared with maladaptive coping (X = 3.57, SD = 0.86).

## Discussion

To our knowledge, this is the first study worldwide to examine the psycho-social correlates of the COVID-19 pandemic lockdown in a group of veterans with PTSD.

At the second assessment, the intensity of the overall PTSD symptoms and of all four PTSD symptom clusters significantly decreased compared with the first assessment. The finding can be explained in several ways. During the lockdown, most participants were exposed to stressors that were mild to the extent that they do not meet the Criterion A of the PTSD diagnosis according to DSM-5 (32). All participants were exposed to restrictive measures, but very few were infected or self-isolated. Previous research clearly indicated that more intense exposure predicted a higher risk of mental health problems (2,43-46).

Disasters are usually categorized into three types: natural disasters, human-made – nonintentional, and human-made – intentional acts (47). The stressors that the participants experienced during the lockdown classify as stressors related to natural disasters. This type of stressors has the lowest traumatic potential. Contrary to this, the highest risk for mental health consequences is associated with interpersonal violence (48,49).

Stressful experiences to which the participants were exposed during the lockdown differ from the initial traumatic war-related stressors. Therefore, they had a lower potential for reactivation or re-traumatization (ie, PTSD exacerbation after the experience of a new traumatic event). War-related stressors are central adverse events to the life story and narrative identity of the majority of veterans. The event centrality concept refers to an individual's interpretation of how central a negative event is to the person's life story (50) and is one of the most substantial factors associated with PTSD (9,51). The stressful experiences related to the COVID-19 pandemic appeared to be far from the central negative event to the participants' life and identity.

Almost all participants followed the protective measures entirely or mostly. Some were even satisfied with the opportunity to practice a greater extent of social distancing. This result is in accordance with recent findings among veterans with PTSD in the USA who seemed unfazed by the COVID-19 restrictions as they had been practicing social distancing (ie, avoidance) for years (52). The most frequently used coping strategies in our sample were acceptance, self-distraction, and emotional support. Similar results were found among health and social workers (53) and students (54). Accepting that the stressor is happening and learning to live with it could be viewed as an adaptive mechanism in a global pandemic. There is evidence that acceptance is a common coping strategy in situations with low controllability (53). Although self-distraction is considered to be a dysfunctional coping strategy, in the context of significant restrictions in everyday life and the overflow of pandemic-related information it might have positive short-term effects. However, the relationship with coping and long-term mental-health outcomes in the COVID-19 pandemic is yet to be investigated. The participants used emotion-focused and problem-focused coping rather than dysfunctional coping. This finding may at first seem surprising as research has repeatedly shown that combat veterans with PTSD use a non-adaptive and avoidance coping style (14-16). As stressors during the lockdown appeared not to have a re-traumatizing effect on most participants, they possibly could engage in more adaptive strategies in dealing with restrictive lockdown measures. The treatment effect can partly explain this finding as previous research revealed that an increase in active and decreased avoidant coping during PTSD treatment incrementally predicted lower PTSD symptom severity (15,55).

Veterans are a homogeneous group with a highly developed group identity. They usually participate in humanitarian activities, continuing their mission to protect the citizens from danger (“combat mode”) and regain full social acknowledgment. Following the restrictions imposed during the lockdown is in line with the described social role and may also be viewed as a form of military subordination. The lack of social acknowledgment predicts the development and worsening of PTSD (56) and the lack of confidence in authorities, and has the most substantial impact on risk perception during disasters (57).

The impact of PTSD treatment between two assessments certainly has to be discussed. Participants have been in treatment for their PTSD symptoms for 16 years on average, implying that they mostly suffer from chronic, usually treatment-resistant, PTSD symptoms with more severe posttraumatic symptoms (ie, complex PTSD) (23). Most of the participants continued to be treated as usual. All participants experienced a decrease in the level of overall PTSD symptoms regardless of the treatment options, implying that the treatment modality did not significantly affect the level of the PTSD symptoms. Despite the treatment, all participants still fulfilled the criteria for PTSD at the second assessment. The results are in line with previous studies, suggesting a poor recovery rate and symptom improvements among patients treated in specialized centers for war-related PTSD several years after the war (58). During the lockdown, all of the participants who received psychopharmacological treatment continued to receive it. Even in circumstances of short-term, temporary disruptions and obstacles, treatment adherence certainly contributed to the mental health and prevented symptoms worsening.

The study limitations are the relatively small sample, which increases the risk of type II errors, and the sample homogeneity. The results cannot be generalized to individuals who are non-help-seeking, non-veteran, or female. Further, self-reports and mixed methodology (telephone vs face-to-face) entail the risk of a reporting bias; thus, causality cannot be determined. Nevertheless, the study is important since it contributes to the body of research as there is still a lack of studies on the impact of the COVID-19 pandemic lockdown on the mental health and psycho-social well-being of the general population and vulnerable groups. Second, the results are important as veterans with pre-existing PTSD have been identified as a high-risk group for various mental health problems during disasters.

As the COVID-19 pandemic is often referred to as a “marathon, not a sprint,” further research is needed to assess the long-term psychopathology trajectories. Future studies should investigate possible long-term mental health consequences of recent adversities, their nature and their extent. It is also important to explore potential mitigating factors in the mental health of the general population and vulnerable groups, such as veterans suffering from long-term posttraumatic problems.
